# Potential relationship between gut microbiota and animal diarrhea: a systematic review

**DOI:** 10.3389/fmicb.2025.1637331

**Published:** 2025-07-24

**Authors:** Yuxin Zhang, Yonggui Ma, Youchao Qi

**Affiliations:** ^1^College of Agriculture and Animal Husbandry, Qinghai University, Xining, China; ^2^Key Laboratory of Medicinal Animal and Plant Resources of Qinghai Tibetan Plateau, Qinghai Normal University, Xining, China; ^3^Academy of Plateau Science and Sustainability, Qinghai Normal University, Xining, China

**Keywords:** diarrhea, gut microbiota, metabolites, treatment, prevention

## Abstract

Diarrhea poses a significant challenge to the growth of the livestock industry by decreasing the productivity and increasing mortality rates in animals. Several factors such as bacteria, viruses, parasites, and stress have been identified as potential contributors to diarrhea. The gut microbiota, a complex micro-ecosystem consisting of trillions of microorganisms such as bacteria, fungi, and viruses, plays a key role in host metabolism, immunity, and nutrient absorption. The gut microbial homeostasis is essential for the intestine to perform physiological functions that maintain the host health. Conversely, gut microbial dysbiosis can lead to the development of various diseases. Recent research has highlighted that gut microbial dysbiosis is a driving factor in the animal diarrhea. Consequently, maintaining the gut microbial homeostasis has become a key focus for the prevention and treatment of diarrhea. This review examines the composition, metabolites and functions of gut microbiota as well as the causes of diarrhea and the alterations in gut microbiota during diarrhea. Furthermore, this review provides insights for future research in this field, especially for alleviating animal diarrhea from gut microbial perspective.

## Introduction

**FIGURE 1:**
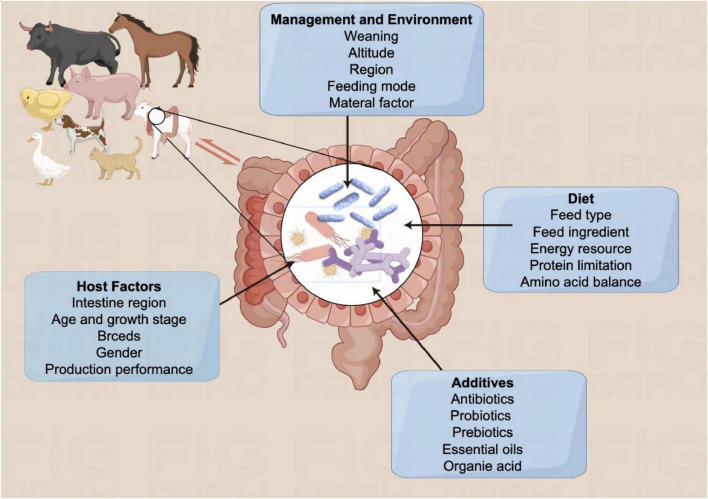
Bidirectional effects between animal and gut microbiota. Gut is an important immune organ of the animal organism, in which gut microbiota can be affected by various factors, such as host factors, additives, diet, management and environment etc.

Intestine is the largest immune organ in the body, and the gut microbial balance is crucial for overall health. These microorganisms, with a significant portion inhabiting the gastrointestinal tract, exert pivotal role in the regulation of physiological functions (Wei et al., 2025). Numerous factors contribute to the imbalance of gut microbiota, including management practices, environmental conditions, diet, additives, and host-related factors (Figure 1). Developing a mutually beneficial connection between the host and gut microorganisms is essential for mucosal immunity and thwarting pathogen colonization (Du et al., 2023).

Previous studies showed the importance of early manipulation of gut microbiota in young animals, especially during the postnatal and early weaning stages, to enhance immunity. Moreover, the prompt colonization of gut microbiota plays a vital roles in developing intestinal barrier function and the immune system maturation (Torow and Hornef, 2017). However, gut microbial dysbiosis may cause various diseases such as neurological, cardiovascular, gastrointestinal issues, and even cancer can arise. Diarrhea is a common consequence of gut microbial dysbiosis, affecting animal health. Therefore, effective preventive measures to reduce diarrhea incidence in animals are crucial in practical production.

Animal diarrhea is a prevalent issue in modern large-scale animal husbandry, affecting various species, particularly young animals, and posing a significant threat to animal health and productivity. Research indicated that young animals such as calves, piglets, and lambs are more susceptible to diarrhea because of their immature immune systems and low resistance levels. In large-scale pig farms, piglet diarrhea rates can exceed 50%, causing a mortality rate of at least 15%. Research conducted by the National Dairy Animal Health Surveillance Program indicates that diarrhea accounts for 57% of deaths among weaned calves, leading to a 38% reduction in net income (Zhang et al., 2022).

Diarrhea in newborn animals can hinder growth and decrease reproductive and milk production in later stages (Du et al., 2023). To address this issue, practitioners employ various strategies to prevent and manage diarrhea. However, China has implemented a policy banning the use of antibiotics in animal feed. However, the complexity of the pathogenesis of diarrhea poses a challenge for effective prevention and control of diarrhea outbreaks. Diarrhea could be divided into two main categories: infectious and non-infectious. Non-infectious factors encompass causes such as stress from weaning, environmental pressures, and mycotoxicosis. Conversely, infectious factors are linked to pathogenic microorganisms, including bacteria, viruses, and parasites.

Presently, research on the causes and mechanisms of diarrhea has demonstrated the potential for reducing incidence through early interventions such as probiotics, prebiotics, herbal additives, and polysaccharides to maintain a balanced gut microbiota (Saha et al., 2024; Yang et al., 2024). Furthermore, fecal microbiota transplantation (FMT) has demonstrated effectiveness in alleviating diarrhea. This review provides a comprehensive analysis of various etiologies and mechanisms of diarrhea, as well as the strategies for modulating gut microbiota and their potential mechanisms, aiming to offer valuable insights for prevention and control of diarrhea.

### Gut microbiota

#### Composition of the gut microbiota

The gut microbiota, a diverse microbial community consisting of bacteria, fungi, archaea, viruses, and protozoa, plays a key roles in maintaining the intestinal structural integrity, immune function, and metabolic balance. Microbial density and diversity increase along the gastrointestinal tract due to varying levels of gastric acid, nutrients, oxygen, and antimicrobial peptides. Recent research indicates a 1:1 ratio of microorganisms to human cells in the body, with the majority residing in the colon (Jordan et al., 2019). Studies on ducks, broilers, laying hens, and piglets have indicated that livestock and poultry gut microbiota are primarily composed of Phylum *Firmicutes* and *Bacteroidetes*, with unique variations at the genus level (Wei et al., 2025; Altintas et al., 2025). Monogastric animals like pigs, chickens, and rabbits typically have dominant genera such as *Anabacterium, Clostridium, Bifidobacterium, Lactobacillus*, and others, with species abundance influenced by factors such as geography, diet, and nutrition (Ley et al., 2008; Sergeant et al., 2014).

#### Mechanism of gut microbiota in maintaining intestinal homeostasis

**FIGURE 2:**
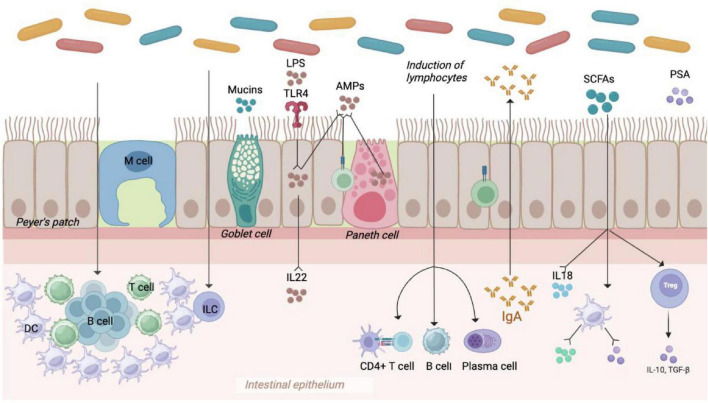
Gut microbiota in the intestinal barrier. Gut barriers include microbial barriers (composed of abundant intestinal flora), mechanical barriers (composed of multifunctional intestinal epithelial cells), and immune barriers (composed of immune cells and cytokines, etc.), which work together to maintain intestinal homeostasis.

The intestinal barrier consists of four primary components: the microbial barrier, mechanical barrier, chemical barrier, and immune barrier (Figure 2). The microbial barrier, primarily composed of anaerobic bacteria, plays a crucial role in resisting the colonization and growth of harmful pathogens within the intestine. Beneficial gut bacteria enhance the microbial barrier by tightly binding to intestinal epithelial cells, outcompeting pathogenic bacteria for nutrients, and producing protective substances like organic acids and antimicrobial peptides. The mechanical barrier, formed by intact intestinal mucosal epithelial cells and tight junctions, acts as a physical defense against bacterial invasion. Beneficial gut microbiota supports the mechanical barrier by promoting epithelial cell development, aiding in mucosal repair, and facilitating mucin synthesis and secretion. Mucins, large glycoproteins secreted by intestinal cells, create a protective barrier that hinders the passage of irritants, toxins, and pathogens, safeguarding intestinal epithelial cells from damage. *Bifidobacterium* and *Lactobacillus* are particularly important for maintaining the integrity of the intestinal mucosal barrier, a key component of the body’s defense system against external antigens. Compromise of this barrier can lead to systemic inflammation. Various components, including gastric acid, bile, enzymes, and organic acids produced by normal gut microbiota, contribute to the chemical barrier. The immune barrier involves intestinal epithelial cells, immune cells, and molecules distributed throughout the mucosa and gut-associated lymphoid tissue (GALT). These barriers work together to maintain intestinal homeostasis. Within the intestinal mucosa, Peyer’s patches, innate and adaptive immune cells, dendritic cells, macrophages, antimicrobial peptides, and immunoglobulin A (IgA) are involved in immune defense mechanisms. Intestinal lymphoid tissues can produce cytokines and antimicrobial peptides to protect against microbial invasion. Additionally, some microbial metabolites have been shown to inhibit the virulence factors of pathogens such as *Salmonella*, thereby contributing to the maintenance of a healthy intestinal ecology (Antunes et al., 2014).

Gut microbiota are essential for maintaining the intestinal barrier and promoting lipolysis (Round et al., 2010; Olszak et al., 2012). Moreover, they are also key players in the development and function of both innate and adaptive immune systems. Notably, gut microbiota has also been shown to be closely related to the intestinal immune maturation. Commensal bacteria in the gastrointestinal tract contribute significantly to the development of the mucosal immune system, which helps maintain the integrity of the intestinal barrier and triggers a natural immune response by recognizing specific receptors. The interaction between innate immunity and gut microbiota is crucial for shaping adaptive immunity (Lundin et al., 2008; Watanabe et al., 2012). Studies indicated that gut microbiota influences the function of dendritic cells (DCs), which are crucial for activating T cells and promoting immune responses (Tan and O’Neill, 2005). Intestinal DCs can drive the differentiation of T- helper cells into subsets that secrete specific cytokines, thereby activating other immune cells like B cells or macrophages. Several research has revealed the symbiotic relationship between microbial communities and intestinal immune cells. Gut microbial dysbiosis can impact the activation of Toll-like receptors (TLRs), decrease the secretion of soluble immunoglobulin A, and modify mucosal innate immunity. Gut microbial dysbiosis can also hinder the differentiation of T cell subtypes and the production of intestinal epithelial cytokines, leading to reduced adaptive immune responses in the intestinal mucosa (Watanabe et al., 2012). The gut microbiota plays a vital role in stimulating both innate and adaptive immunity, such as the production of cytokine IL-22 that enhances the production of antimicrobial peptide AMP by acting on epithelial cells. Furthermore, pattern recognition receptors (PRRs) are a key component of innate immunity, which could recognize and response to various microbial components, such as peptidoglycan, lipopolysaccharide, and formylated peptides (Rooks and Garrett, 2016). The initiation of pattern recognition receptors (PRRs) leads to the secretion of chemokines, cytokines, and apoptotic factors via a signaling cascade involved in disease mechanisms. For instance, the microbial polysaccharide PSA is capable of directly entering the circulatory system by the host intestinal epithelial cells (Watanabe et al., 2012).

In the circulatory system, PSA facilitates the interaction between dendritic cells (DCs) and T cells through MHC class II molecules and T cell receptors (TCRs) to inhibit inflammation. Within the host’s genito-urinary tract, host defense peptides (HDPs) enhance the release of pro-inflammatory cytokines by immune cells in the host via the TIFA/TRAF6/NF-κB signaling pathway. Additionally, formyl peptides modulate the inflammatory response of neutrophils by binding to formyl peptide receptor 1 (FPR1), which is expressed on neutrophils (Blyth et al., 2020).

#### Diarrhea induces alterations in the gut microbial composition

Gut microbial dysbiosis poses a threat to the host’s health, potentially leading to systemic diseases like obesity, diabetes, and cancer in addition to diarrhea. Previous studies indicated that diarrhea can significantly reduce the gut microbial diversity and alter their taxonomic composition, causing a decrease in beneficial bacteria. Various studies have explored the specific changes in gut microbiota during animal diarrhea, offering insights for preventing and treating diarrhea by targeting the gut microbiota (Li et al., 2022). In piglets, the most common genera in the gut microbiota include *Lactobacillus* spp., *Shigella* spp., *Enterococcus* spp., *Bacteroides* spp., and *Clostridium* spp. Healthy piglets typically exhibit a predominance of *Lactobacillus*, while diarrheic pigs show increased levels of *E. coli-Shigella* and *Enterococcus*, and decreased levels of *Bacteroides*. The composition of the microbiome varies across different parts of the intestine, with *Lactobacillus* being most prevalent in the ileum, and *Clostridium* and *Bacteroides* being more common in the rectum (Gryaznova et al., 2022). At the phylum level, healthy calf herds exhibit high abundances of *Firmicutes*, *Bacteroides*, *Aspergillus, Clostridium*, and *Actinobacteria.* Diarrhea is associated with an increase in *Aspergillus* and *Clostridium*, and a decrease in *Bacteroides* and *Actinobacterium* (Shen et al., 2023). Furthermore, significant alterations in the alpha and beta diversities of the gut fungal community have been observed in diarrheic horses, leading to marked changes in taxonomic composition. Although healthy and diarrheic horses share major fungal phyla like *Neocallimastigaceae, Ascomycetes, and Basidiomycota*, the relative abundance of these phyla differs between the two groups. The composition of dominant fungal genera in diarrheic horses differed significantly from that of healthy horses. Metastats analysis revealed a significant decrease in various fungal phyla during the period of diarrhea. Furthermore, a total of 175 fungal genera were identified in the gut fungal community of healthy and diarrheic horses, with 4 fungal genera showing a significant increase and 171 bacterial genera showing a significant decrease during diarrhea. Among the reduced bacteria, 74 fungal genera were completely absent from the intestine (Li et al., 2022).

#### Diarrhea

The etiology of diarrhea can be classified into infectious and non-infectious diarrhea. Moreover, it can be classified as secretory, exudative, hyperactive, osmotic, and malabsorptive diarrhea depending on the underlying mechanism (Table 1).

**TABLE 1:** Classification of diarrhea and potential mechanisms.

Type	Mechanism	Example
Secretory diarrhea	Intestinal secretion of large amounts of fluid exceeds the absorptive capacity of the intestinal mucosa	Massive watery diarrhea caused by Vibrio cholerae exotoxin
Exudative diarrhea	Inflammation of the intestinal mucosa exudes large amounts of mucus, pus and blood resulting in diarrhea	
Osmotic diarrhea	It is caused by elevated osmotic pressure of the intestinal contents, which prevents the absorption of water and electrolytes from the intestines.	Diarrhea caused by taking salt laxatives or mannitol
Dynamic diarrhea	Shortened retention time of intestinal coeliac due to hyperperistalsis, not fully absorbed	
Malabsorptive diarrhea	Caused by reduced or impaired absorption in the intestinal mucosa	*Giardia*, pathogenic *Escherichia coli* (EPEC), rotavirus

#### Non-infectious diarrhea

In animal production and management, various factors including malnutrition, environmental changes, autoimmune diseases, and management practices cause stress in animals in addition to the infections of pathogenic bacteria. This stress can disrupt intestinal homeostasis and cuase diarrhea. For instance, weaning stress in piglets can suppress their immune response, affect digestive physiology, and reduce the activity of digestive enzymes, leading to diarrhea. Other causes of diarrhea include metabolic issues due to an imbalanced nutritional ratio in feed and the presence of mycotoxins. Young animals, characterized by underdeveloped body functions, weak digestive capabilities, poor gastrointestinal function, and low resistance, are particularly susceptible to bacterial, viral, and parasitic infections that can induce diarrhea. Secretory diarrhea, a prevalent type of diarrhea, occurs when there is an imbalance between the absorptive and secretory functions of the intestines. This imbalance results in excessive fluid secretion, primarily driven by Cl^–^ secretion, which exceeds the absorptive capacity of the intestines, leading to increased water content in the feces. Secretory diarrhea commonly arises from infections caused by pathogenic bacteria and viruses, exposure to allergens, disruption of bile acid homeostasis, or as a side effect of various medications (Keely and Barrett, 2022).

#### Infectious diarrhea

The etiology of infectious diarrhea is diverse, typically involving bacteria, fungi, viruses, and parasites. These intestinal pathogens can directly impact epithelial ion transport processes and barrier function, causing diarrhea. Moreover, they may indirectly cause intestinal dysfunction by inflammation, neuropeptides, or loss of absorptive surfaces in the gut, causing diarrhea. For instance, pathogens like the intestinal parasite *Giardia flagellata* can result in the loss of the absorptive surface of the brush border and diffuse shortening of the villi. Similarly, enteropathogenic *Escherichia coli* (EPEC) can lead to the loss of intestinal microvilli, reducing the surface area for nutrient absorption and causing increased osmotic pressure and malabsorption of intestinal contents. Various bacteria, viruses, and parasites can induce diarrhea in animals, as illustrated in Table 2. Notably, different pathogens exhibit varying levels of pathogenicity in animals, resulting in a diverse range of clinical symptoms. For example, *E. coli* is known to trigger diarrhea in pigs, cattle, and sheep, whereas chickens infected with *E. coli* typically display symptoms such as hepato-pancreatitis and pericarditis.

**TABLE 2:** Common infectious diarrhea pathogens in different animals.

Type of pathogen	Pig	Cow	Goat	Chicken
Bacteria	*Escherichia coli* (*E. Coli*) *Salmonella* Swine dysentery Dense Spirochete	*Escherichia coli* (*E. Coli*) *Salmonella* Pasteurella multocida Campylobacter jejuni	*Escherichia coli* (*E. Coli*) *Salmonella* Mildew Clostridium difficile Streptococcus	*Escherichia coli* (*E. Coli*) *Salmonella* Pasteurella
Viruses	Porcine epidemic diarrhea virus Coronavirus Rotavirus Swine Fever Pseudorabies	Bovine viral diarrhea virus Coronavirus Rotavirus	Epidemic diarrhea virus Rotavirus	Avian Influenza virus Newcastle disease virus Adenovirus Avian pox virus
Parasites	*Coccidia* *Roundworm* Packed worms *Colonic pouch ciliates*	*Coccidia* *Cryptosporidium* Giardia lamblia protozoan flagellate	*Coccidia* *Cryptosporidium* Cestodes Nematodes Schistosoma japonicum	*Coccidia* Trematode worm Ascaris lumbricoides Bile duct worms

#### Bacteria and diarrhea

*Escherichia coli*, *Shigella*, *Salmonella*, *Campylobacter*, *Clostridium difficile*, and *Aeromonas* are the main pathogens that cause diarrhea. Previous studies indicated that the rapid onset of Enteropathogenic *Escherichia coli* (EPEC)-induced diarrhea may be attributed to a direct impact on intestinal epithelial ion transport processes (Croxen et al., 2013). Invasive pathogens like *Shigella* and *Salmonella* species lead to inflammatory diarrhea characterized by fever and the presence of polymorphonuclear cells (PMN) in feces. PMN could regulate absorption through cytokine secretion and induce diarrhea by secreting adenosine precursors, which activate CFTR (cystic fibrosis transmembrane conductance regulator). Additionally, *Clostridium difficile* and *Clostridium rotundum* infections indirectly affect ion transport through cytokine secretion and by stimulating enteric nerves via neuropeptides. *Escherichia coli* are common opportunistic pathogens in the large intestine and are typically non-pathogenic. However, the acquisition of virulence-encoding genes can render them pathogenic to the host. Pathogenic *E. coli* strains can be categorized as enterotoxin-producing (ETEC), enteropathogenic (EPEC), adherent-invasive (AIEC), entero-invasive (EIEC), diffuse adherent (DAEC), enteroaggregative (EAEC), and Shiga toxin-producing (STEC) strains, including enterohemorrhagic (EHEC). Some of these types of *E. coli* can cause diarrhea in various animals, such as ETEC in piglets, calves, and lambs, EPEC in rabbits, dogs, cats, pigs, calves, and lambs, and Shiga toxin-producing *E. coli* in calves and piglets (Aydin et al., 2022). So far, EAEC, EIEC, and DAEC have not been identified as pathogens causing animal diarrhea (Croxen et al., 2013). Piglets infected with *E. coli*-associated diarrhea typically exhibit feces that are white-gray or yellow, often characterized by yellow bubbles and a fishy odor. Yellow diarrhea is observed in piglets during the first week of life, referred to as early onset, while white diarrhea is more common in piglets aged between 1 and 4 weeks, known as late onset. Infection with enterotoxin-producing *Escherichia coli* (ETEC) F4 + disrupts the intestinal epithelial barrier, decreases the number of goblet cells, and lowers the total short-chain fatty acid (SCFA) levels in the colon (López-Colom et al., 2020). In calves, *E. coli* infection induces diarrhea by disrupting tight junctional proteins in the intestines and releasing cell-damaging toxins that harm the small intestinal mucosa, leading to mucosal inflammation (Wu et al., 2022). Invasion of pathogenic bacteria triggers the disruption of tight junction proteins and activation of inflammatory signaling pathways, such as I-kappa-B-alpha, mitogen-activated protein kinase, and nuclear factor-κB. The invasion additionally prompts small intestinal epithelial cells to produce IL-8, IL-6, and TNF-α, which causes an inflammatory response in calves (Gandhar et al., 2022).

Furthermore, weaning stress can increase serum cortisol levels, potentially compromising cellular immunity and suppressing various non-specific immune responses, thereby increasing susceptibility to diseases (Kim et al., 2020). This stress also leads to elevated blood levels of neutrophils, TNF-α, IL-6, and IL-1β, alongside increased expression of apoptotic and pro-inflammatory factors, including IL-1β, IL-8, interferon-gamma, TNF-α, and TLR-4. Collectively, these factors trigger an inflammatory response in the calf intestine (O’Loughlin et al., 2011). Infection of lambs with *E. coli* NMGCF-19 results in severe diarrhea and neurological disorders, research on mice models showed a significant decrease in ZO-1 and occludin expression in mice brain tissue. Furthermore, NMGCF-19 infection induces upregulation of pro-inflammatory cytokines TNF-α, IL-6, IL-1β, and IL-18, higher expression levels of HMGB1, and activation of the TLR2/TLR4/MyD88 and NLRP3 inflammasome pathways (Wang et al., 2020).

*Salmonella*, a gram-negative intracellular pathogen, is classified based on its lipopolysaccharide (LPS) O antigen and *flagellar* H antigen, with over 2,600 serotypes identified to date (Lamichhane et al., 2024). It is a significant cause of infectious diarrhea and a prominent zoonotic pathogen worldwide, posing significant public health risks and economic losses in livestock (Quach et al., 2022). *Salmonella* infections typically spread through fecal-oral contamination among livestock, often originating from contaminated feed, water, equipment, and inadequate hygiene practices among farm workers. Furthermore, certain serotypes, such as Salmonella Dublin, can be transmitted via aerosols in confined captive calves. Studies indicate that *Salmonella*-induced diarrhea may result from the inhibition of the Notch signaling pathway, which leads to a transition in intestinal cells from absorptive to secretory types, ultimately diminishing cellular absorption and resulting in diarrhea (Diepold and Armitage, 2015). In piglets, *Salmonella* infection is characterized by persistent watery feces, which are often yellowish-green or dark brown, and contain necrotic intestinal mucosal tissue, emitting a foul odor. This condition can lead to growth retardation and, in severe cases, mortality. In sheep, *Salmonella* infections are frequently associated with sheep enterotoxemia, exhibiting the highest infection and mortality rates in lambs, along with severe diarrhea in pregnant ewes. The *Salmonella* type III protein secretion system (T3SS) plays an important role in bacterial invasion of host cells and subsequent replication (Diepold and Armitage, 2015). The invasion process involves the translocation of effector proteins such as SipA, SipB, SipC, and SopB into the host cell, leading to cytoskeletal remodeling that facilitates *Salmonella* cell invasion (Lou et al., 2019). SipA is the key protein involved in bacterial invasion, with SipB forming protein complexes with SipC to promote host cell invasion by *S. typhimurium* (Myeni et al., 2013). Structural components of T3SS-1 are essential for *Salmonella* invasion of bovine and porcine ileal mucosa, with specific effector proteins (SipA, SipC, SopE/E2, SopB, SopD, and SptP) playing roles in manipulating actin dynamics (Shi et al., 2025). These effector proteins can mimic eukaryotic guanine nucleotide exchange factors (GEFs) or produce secondary messengers to activate host GEFs and Rho family GTPases, ultimately affecting actin assembly and cellular structure (Singh et al., 2020). Additionally, SptP acts as a mimic of host GTPase-activating proteins to deactivate Cdc42 and Rac1, reversing signaling pathways initiated by SopB, SopE, and SopE2 (Ly and Cyert, 2017). *Salmonella* infections occur through fecal-oral transmission, overcoming various intestinal barriers to interact with the intestinal epithelium and penetrate host tissues (Gopinath et al., 2012; Barbara et al., 2021). Previous studies showed that *Salmonella* attaches to epithelial tissues and disrupts cellular junctions, ultimately causing intestinal inflammation and diarrhea (Wang et al., 2022; Zhang et al., 2012). Therefore, preserving the integrity of epithelial tissue could potentially reduce the pathogenicity and severity of *Salmonella* infection.

The transmission of *Clostridium difficile (C. difficile)* occurs via the fecal-oral route, leading to a spectrum of symptoms ranging from asymptomatic cases to severe diarrhea, which can culminate in life-threatening conditions. The normal gut microbiota serves as a protective barrier against *C. difficile infection* (CDI). Disruption of this balance allows *C. difficile* to colonize the large intestine and become predominant. CDI is increasingly recognized as a significant cause of enteritis in neonatal piglets, potentially resulting in growth retardation, delayed weaning, and mortality in swine. Additionally, it can cause colitis in large birds such as ostriches. The primary virulence factors are two toxins, A (TcdA) and B (TcdB). Research has indicated that toxin A primarily drives the pathogenic process, with toxin B exerting its effects following the tissue damage caused by toxin A. These toxins disrupt the intestinal epithelial cytoskeleton and tight junctions, increase fluid secretion, promote neutrophil adhesion, and ultimately compromise the integrity and function of the intestinal barrier (Czepiel et al., 2019). Most *C. difficile* strains isolated from animals also produce a binary toxin (*C. difficile* transferase) that enhances virulence. Toxins are transported to the cytoplasm where they inactivate the Rho family of GTPases. Rho proteins are crucial for actin polymerization and maintaining cytoskeleton stability. When Rho proteins are inactivated, cytoskeletal stability diminishes, exacerbating the inflammatory process. In severe cases, piglets infected with *C. difficile* may develop watery, yellow, pasty, non-hemorrhagic diarrhea, with micro-ulcers and pseudomembranes on the intestinal mucosa.

*Campylobacter jejuni*, a gram-negative bacterium with a spiral or rod-shaped form, is recognized for causing fever, bloody diarrhea, and inflammatory diarrhea, similar to *Salmonella* and *Shigella* (Giallourou et al., 2018). *Aeromonas*, a genus of anaerobic gram-negative bacillus, produces oxidizing enzymes, catalase, and other exoenzymes. *Clostridium perfringens* infection in piglets leads to pink and reddish-brown feces, known as necrotizing enteritis, with high mortality rates in 1-week-old piglets. Swine dysentery and *Lawsonella intracellularis* can also cause diarrhea in pigs (Burrough, 2017; Campillo et al., 2021).

#### Parasitic infestation and diarrhea

Intestinal parasites represent a significant cause of diarrhea, often underestimated in terms of their public health implications. These parasites can profoundly affect the intestinal physiology and immune system of the host, leading to intestinal damage and disrupting the relationship between gut microbiota and host immunity. Common parasites, including *Coccidia*, *Cryptosporidium*, *Roundworms*, *Whipworms*, and *Colonic pouch ciliates*, can induce diarrhea by compromising the intestinal mucus and epithelial barriers. Furthermore, they impact both the innate and adaptive immunity of the host while altering the gut microbiota, thereby affecting the overall intestinal environment.

For instance, unicellular protozoa of the phylum *Apicomplexa*, such as *Eimeria* spp., cause severe infections in livestock, particularly in poultry and cattle (Morgoglione et al., 2020). Avian coccidiosis alone has caused over $3 billion in economic losses globally to the poultry industry (Balta et al., 2021). These protozoa not only affect gastrointestinal function and resident microbial composition but also contribute to mucosal damage, which can predispose individuals to necrotizing enterocolitis and subsequent bacterial infections like *Salmonella typhimurium* and *Clostridium perfringens* (Macdonald et al., 2019; McKnight et al., 2019). In calves, *Eimeria bovis* and *Eimeria kewi* are common and highly pathogenic, with *Eimeria bovis* inhibiting NF-κB activation, impairing gene expression of immunomodulatory molecules, regulating apoptosis and cholesterol metabolism, and reducing cytoskeletal integrity. These effects lead to acute hemorrhagic diarrhea, dehydration, weight loss, and a significant decrease in growth rate (Velásquez et al., 2021).

Following infection with *Cryptosporidium*, epithelial cells release pro-inflammatory cytokines and chemokines to the infection site, potentially causing increased epithelial permeability, impaired intestinal absorption, and increased secretion. In cases of intestinal cryptosporidiosis, villous atrophy and crypt hyperplasia have been identified as factors contributing to impaired monosaccharide and glucose-Na absorption (Certad et al., 2017). It has been proposed that the down-regulation of specific key components of tight junctions (TJ) and adherents junctions (AJ) to disrupt barrier function may play a significant role in diarrhea resulting from *Cryptosporidium* infection (Kumar et al., 2018). *Cryptosporidium* is commonly found in conjunction with other pathogens, such as *Cryptosporidium* minis, *Cryptosporidium* bovis, *Cryptosporidium* anserineum, and *Cryptosporidium* ruber. *Cryptosporidium* infection induces cytoskeletal changes that control actin reorganization and the insertion of channel/transporter proteins (Meremikwu et al., 2012). Numerous studies have indicated that the infection of intestinal and biliary epithelial cells necessitates host cell actin polymerization and cytoskeletal remodeling (Färnert et al., 2014). This polymerization involves the actin branching and nucleation mechanisms of the Arp2/3 complex protein (Snow et al., 2015). Multiple signaling pathways have been demonstrated that regulate actin reorganization and the internalization of *Cryptosporidium*, including PI3K, guanine exchange factor, and CDC42- and c-Src-dependent activation of corticosterone (Meremikwu et al., 2012). *Cryptosporidium* activates NF-κB to inhibit apoptosis in biliary epithelial cells, which is crucial for cell survival. NF-κB plays a role in activating various intracellular survival signals, including the C-myc oncogene (Carneiro et al., 2010). A study conducted on *Cryptosporidium*-infected human (Caco-2) and bovine (MDBK and NBL-1) epithelial cells *in vitro* revealed disruption of occluded ZO-1 and fragmentation of the nucleus during infection (Roca-Feltrer et al., 2010).

*Giardia* infection disrupts, reduces expression, and relocates tight junctions and cytoskeletal proteins such as ZO-1, claudin-1, F-actinin, and α-actinin, resulting in increased intestinal permeability and decreased trans-epithelial resistance. This disruption can lead to paracellular leakage and exudative diarrhea. *Giardia flagellates* interfere with tight junctions without penetrating the epithelium, causing secretory diarrhea characterized by elevated chloride ion concentrations, loss of absorptive function (including microvilli atrophy and increased cell death), heightened secretion (due to epithelial barrier disruption), and inadequate disaccharidase activity and nutrient malabsorption stemming from the loss of the microvillar brush border and villi atrophy, ultimately resulting in osmotic diarrhea.

#### Viruses and diarrhea

Common clinical viruses that can cause diarrhea in animals include rotavirus, coronavirus, and epidemic diarrhea virus. The mechanisms underlying viral diarrhea are primarily secretory and exudative. Rotavirus predominantly affects the small intestine of pigs, damaging intestinal villi and resulting in acute diarrhea, dehydration, and mortality in piglets (Chepngeno et al., 2020). In calves, bovine rotavirus (BRV) can induce acute diarrhea within 12–24 h post-infection, with substantial amounts of the virus excreted in feces, thereby facilitating transmission (Jang et al., 2019). The infection mechanism involves viral replication in small intestinal epithelial cells, leading to destruction of mature cells, activation of the enteric nervous system, and secretion of viral enterotoxin (Kayasaki et al., 2021). Additionally, rotavirus infection affects the diversity, homogeneity, and abundance of gut microbiota. Sheep and alpacas are also susceptible to rotavirus infection, which can result in diarrhea (Rojas et al., 2016; Shabana et al., 2017).

Porcine enteric coronaviruses (PECs) such as PEDV, PDCoV, TGEV, and SADS-CoVare known to cause severe diarrhea in young pigs, posing a significant threat to the global swine industry (Table 3). These viruses primarily target the digestive system of piglets, resulting in symptoms such as weight loss, lethargy, vomiting, loss of appetite, watery diarrhea, and, in severe cases, death. The pathological effects include necrosis and detachment of enterocytes, as well as damage to intestinal villi (Pan et al., 2017; Suzuki et al., 2018).

**TABLE 3:** Classification of porcine enteric coronaviruses.

Viruses	Mortality in neonatal piglets	Pathogenicity for other species	Clinical symptoms
PEDV (Alphacoronavirus)	Almost 100%	No report	Vomiting, watery diarrhea, dehyration, weight loss
PDCoV (Deltacoronavirus)	50–100%	Humans, calves, chickens	Vomiting, watery diarrhea, dehyration, weight loss
TGEV (Alphacoronavirus)	Up to 100%	No report	Dehyration, weight loss, abortion
SADA-CoV (Alphacoronavirus)	More than 90 in pigs ≤ 5 days of age	No report	Acute diarrhea, acute vomiting, acute death

Studies have indicated that PEDV S1 facilitates viral invasion through interaction with EGFR, triggering the EGFR-regulated downstream EGFR/ERK signaling pathway. This cascade results in decreased NHE3 expression, impaired NHE3 migration at the plasma membrane, and ultimately reduced NHE3 activity (Zhang et al., 2023c). The downregulation of NHE3 expression in intestinal epithelial cells is believed to play a significant role in the development of PEDV-induced diarrhea in neonatal piglets. Infection with porcine intestinal coronavirus SeCoV induces autophagy, apoptosis, and innate immune responses, with the intricate interplay between these responses potentially impacting viral replication or modifying specific signaling pathways to induce pathological damage in SeCoV-infected hosts. Coronaviruses can also infect cattle, with bovine coronavirus (BCV) causing calf enteritis in dairy and beef cattle. Animals typically affected are usually aged between 1 day to 3 months and diarrhea occurs within 1–2 weeks after birth, reaching its peak between days 7 and 10. Calves can become infected with BCV via their oral and respiratory routes, exhibiting clinical symptoms around 2 days after infection, which typically persist for a duration of 3–6 days (Hodnik et al., 2020).

Calves, particularly those that do not receive colostrum, are at an increased risk of severe diarrhea due to secondary coronavirus infections. These infections typically originate in the upper portion of the small intestine and subsequently spread throughout the entire small intestine (Vlasova and Saif, 2021). The virus utilizes spiny projections and hemagglutinin glycoproteins to attach to the epithelial cells of the intestine, leading to the fusion of the viral envelope with either the host cell membrane or intracellular vesicles. Following this, the virus is released to replicate and induce cell death via normal secretory mechanisms, ultimately resulting in diarrhea. Bovine viral diarrhea virus (BVDV) is recognized as a causative agent of diarrhea in cattle, with 21 subtypes A (BVDV 1a-u), 4 subtypes B (BVDV 2a-d), and 4 subtypes H (BVDV 3a-d) identified. BVDV has the capability to cross the placental barrier during early gestation, which can lead to persistent infections in calves. Managing these infected calves poses significant challenges, as they continuously harbor and shed the virus throughout their lives, becoming the primary reservoir for BVDV infection. In addition to the aforementioned pathogens, other viruses such as bovine astrovirus, bovine circovirus, bovine norovirus, bovine enterovirus, and bovine microvirus may also contribute to diarrhea in calves. Moreover, BCoV and bovine-like CoV have been identified in various domestic and wild ruminants, as well as in dogs and humans (Vlasova and Saif, 2021).

#### Improving the gut microbiota alleviates symptoms of diarrhea

Diarrhea ranks among the foremost reasons for death in young animals, such as calves, piglets, and lambs. Due to their immature immune systems, these young animals are more vulnerable to deterimental factors such as bacteria, viruses, and stress, which may disrupt the gut microbial balance and result in diarrhea. Therefore, interventions such as dietary supplementation with probiotics, prebiotics, herbal additives, and polysaccharides are effective in maintaining gut microbial balance and alleviating diarrhea. Furthermore, fecal microbiota transplantation (FMT) has also shown promise in reducing the incidence of diarrhea.

#### Probiotics and their metabolites

**FIGURE 3:**
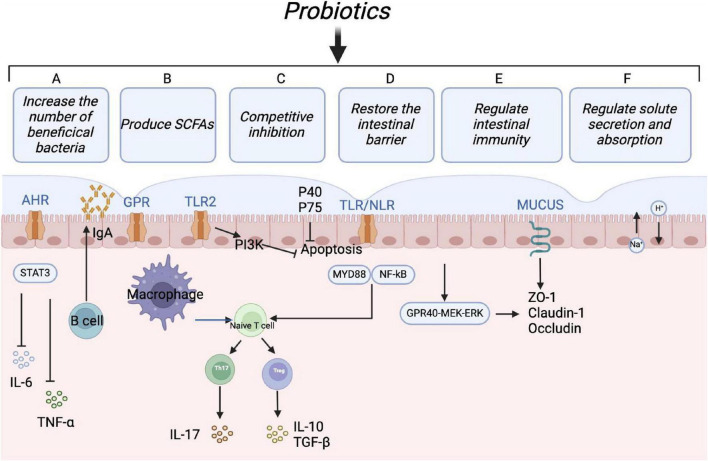
Protective mechanisms of probiotics. Probiotics can maintain intestinal microbial homeostasis in multiple ways: (1) Increasing the relative abundance of beneficial bacteria; (2) regulating T cells differentiation to relieve inflammatory reaction and immunologic derangement; (3) maintaining the intestinal barrier by regulating tight junction proteins; (4) enhancing humoral immune response and inducing anti-inflammatory effects; (5) promoting fluid absorption via the exchange of Na^+^ and H^+^ in epithelial cells, reducing the risk of antibiotic-associated diarrhea.

Probiotics play a key role in regulating the gut microbial homeostasis, enhancing resistance to intestinal pathogens by maintaining a healthy gut microbial balance (Figure 3). They produce metabolites that can inhibit pathogenic bacteria, interfere with microbial virulence gene expression, and regulate host gene expression (Önlü et al., 2025). Additionally, probiotics modulate intestinal immune function and effectively treat pathogen-induced diarrhea. The active ingredients of probiotics include antimicrobials, vitamins, peptides, biosurfactants, extracellular polysaccharides, and enzymes. The structural features on the surfaces of probiotics, including flagella, hyphae, surface layer proteins (SLP), podocarp polysaccharides (CPS), lipophosphatidic acids, and lipopolysaccharides, function as MAMPs that interact with PRRs, such as NLRs and TLRs (Lebeer et al., 2018; Liu et al., 2020).

Probiotics may prevent or treat diarrhea by outcompeting pathogens for nutrients, producing antimicrobial compounds, and inhibiting harmful bacterial colonization (Endt et al., 2010). Research has demonstrated that *Lactobacillus XLTG11* can reduce intestinal pathology by decreasing abundance of LPS, D-LA, and DAO, as well as improving intestinal permeability. This probiotic additionally promotes the expression of proteins involved in water channels and tight junctions, inhibits the TLR4/NF-κB signaling pathway, elevates the levels of anti-inflammatory cytokines, and diminishes the levels of pro-inflammatory cytokines. The content of acetic acid, propionic acid, butyric acid, and total short-chain fatty acids significantly increased after treatment with *Lactobacillus XLTG11*. Compared to the MC group, *Lactobacillus XLTG11* enhanced the gut microbial abundance and diversity and induced changes in the gut microbial composition (Xu et al., 2022). Moreover, the rest of the probiotics listed in Table 4 show some effectiveness in relieving diarrhea and regulating gut microbiota.

**TABLE 4:** The role of probiotics in relieving diarrhea.

Probiotic strains	Mechanism of action	References
*Bifidobacterium BB536*	Reducing the number of *Salmonella* in the intestine or improving the expression of tight junction protein genes ameliorates *Salmonella*-induced intestinal barrier damage in broiler chickens.	(van Baarlen et al., 2011; Wang et al., 2018)
*Lactobacillus FloraMax-B11*	Promote the formation and stabilization of gut microbiota and reduce the infection rate of pathogenic bacteria in newborn chicks.	(Prado-Rebolledo et al., 2017)
*Lactobacillus rhamnosus HN001*	Regulates the composition of the gut microbiota, leading to a significant decrease in potentially harmful bacteria and an increase in beneficial bacteria.	(Toscano et al., 2017)
*Bacillus subtilis*	Interferes with *Staphylococcus aureus* population sensing by secreting antimicrobial peptides and completely inhibits colonization of the pathogen *Staphylococcus aureus*.	(Piewngam et al., 2018)
*Bifidobacterium*	Negative regulation of Vibrio cholerae T6SS gene expression by regulating bile acid metabolism.	(Bachmann et al., 2015)
*Lactobacillus acidophilus La-5*	Expression of the tight adhesion eaeA gene, the adhesion protein espA gene, and the flagellin fllC gene were all reduced by more than 60% in *Escherichia coli* O157:H7.	(Medellin-Peña et al., 2007)
*Lactobacillus acidophilus*	Significantly inhibited the expression of eight virulence genes in Helicobacter pylori virulence islands.	(Ryan et al., 2009)
*Lactobacillus rhamnosus* *Lactobacillus plantarum*	The participation of protein kinase C (PKC) and processes reliant on mitogen-activated protein kinase (MAPK), along with the suppression of cytokine-triggered apoptosis and damage to epithelial cells through a phosphatidylinositol 3-kinase-AKT-dependent mechanism.	(Seth et al., 2008; Anderson et al., 2010; Bron et al., 2012)
*Lactobacillus plantarum*	Enhancement of Epithelial Barrier Function Potentially Achieved by Suppressing the Expression of Tight Junction Proteins and Diminishing ETEC F4-Induced Proinflammatory Cytokines through the Modulation of TLRs, NF-κB, and MAPK Signaling Pathways.	(Wu et al., 2016)
*Lactobacillus acidophilus*	Enhancement of Th1 cell maturation via the activation of Th1 cytokine synthesis through the IFN-STAT3-NF-κB signaling pathway.	(van Zyl et al., 2020)
*Clostridium butyricum*	Improved the chemical and mechanical barriers intestinal in weaned piglets through TLR-2-MyD88-NF-κB signaling.	(Fu et al., 2021)
*Lactobacillus rhamnosus 4B15, Lactobacillus casei 4M13*	Inhibition of LPS-induced production of pro-inflammatory cytokines TNF-α and IL-6 in intestinal cells	(Hakansson and Molin, 2011; Oh et al., 2018)

Probiotics are capable of producing beneficial metabolites, including prebiotics such as oligofructose, inulin, and pectin oligosaccharides. These metabolites function as soluble decoy receptors, which help prevent pathogen colonization and facilitate the elimination of pathogens from the intestine (Rigo-Adrover et al., 2017). Furthermore, metabolites like secreted proteins, indoles, and short-chain fatty acids produced by probiotics play a crucial role in protecting the intestinal barrier. Notably, butyrate synthesized by *Clostridium butyricum* improves the immune function and intestinal barrier by increasing the levels of hypoxia-inducible factor (HIF-1α), mucins, antimicrobial peptides, and IL-22 in the intestines (Pral et al., 2021). Indole-3-lactic acid produced by *Bifidobacterium infantis* and *Lactobacillus rhamnosus* activates aryl hydrocarbon receptors (AhRs), which enhances the expression of CYP1A1. This process ultimately promotes the transcription of IL-22 and the expression of antimicrobial peptides (Ehrlich et al., 2020; Hou et al., 2021). Additionally, the soluble proteins P40 and p75 derived from *Lactobacillus rhamnosus* activate EGFR, promoting IgA secretion and preserving intestinal homeostasis via the EGFR-PIK3-Akt signaling pathway (Wang et al., 2017). The proliferation of short-chain fatty acid-producing microorganisms in the hindgut of weaned piglets reduces intestinal pro-inflammatory factors and enhances the intestinal immune barrier through the MyD88-NF-κB signaling pathway (Tian et al., 2022).

#### Prebiotics and postbiotics

Oligofructose, oligogalactose, oligo xylose, oligo isomaltose, soybean oligosaccharides, and inulin are commonly used prebiotics. Certain microalgae, such as spirulina and other algae, can also function as prebiotics. Additionally, polysaccharides (e.g., Yunzhi polysaccharides, carrageenan polysaccharides with nitrogen), protein hydrolyzates (e.g., casein hydrolyzates, alpha lactalbumin, lactoferrin), and natural plants from vegetables, herbs, and wild plants can be utilized as prebiotics. Postbiotics have garnered significant attention due to their potential applications in functional foods and pharmaceuticals. The International Society for the Science of Probiotics and Prebiotics (ISAPP) has established a standardized consensus definition for postbiotics. According to ISAPP, postbiotics are preparations of non-living microorganisms or their constituents that offer health benefits to the host. These postbiotics can include fully or partially inactivated bacteria, with or without metabolic byproducts. Various terms have been employed in published studies to refer to postbiotics, including inactive probiotic, butylated probiotic, heat-killed probiotic, cell lysate, parabiotic, ghost probiotic, and postbiotic (Salminen et al., 2021). Postbiotic formulations often include microbially produced components such as metabolites, peptides, enzymes, proteins, vitamins, and extracellular polysaccharides (EPS), which contribute to the overall health benefits of postbiotics (Deshpande et al., 2018). Postbiotic forms of probiotics can be produced through optimal application of heat, ultraviolet light, and sonication. Live probiotics may encounter difficulties adhering to the intestinal mucosa due to the barrier posed by the mucosal layer, which limits direct bacterial contact. In contrast, postbiotics can easily penetrate the mucosal layer, posing no risk of infection or transfer of antibiotic resistance genes in vulnerable individuals. This enhances the convenience of dosage maintenance, transport, standardization, and storage of postbiotics (Deshpande et al., 2018). Therefore, utilizing inactivated bacteria as a supplement may present a safer alternative to live probiotics for susceptible populations, such as neonates, potentially aiding in the treatment of gastrointestinal diseases and immune disorders (Ou et al., 2011; De Marco et al., 2018). In their inactivated form, strains like *Lactobacillus* and *Bifidobacterium* exhibit immunomodulatory effects by stimulating innate and adaptive immunity, as well as enhancing the membrane integrity of intestinal epithelial cells (Hirose et al., 2006; Adams, 2010; Sugahara et al., 2017). These effects are mediated through various signaling receptors, including TLR in dendritic cells, intestinal epithelial cells, and other immune cells (Soares et al., 2010).

#### FMT and diarrhea

FMT involves transferring functional microbiota from healthy donors to diseased individuals to restore normal gut microbiota and treat intestinal diseases. Early FMT intervention has been demonstrated to increase growth performance and intestinal barrier function in piglets by promoting symbiotic bacterial colonization and increasing intestinal oxygen concentration (Geng et al., 2018). Previous research indicated that feeding neonatal piglets FMT suspensions from healthy adult pigs can significantly improve growth performance and intestinal barrier function (Zeng et al., 2016). Additionally, probiotics play a key role in maintaining intestinal homeostasis, regulating gut microbiota, and reducing diarrhea. Combining early FMT intervention with probiotics has been demonstrated to enhance growth performance, reduce diarrhea, and improve intestinal barrier function by increasing the abundance of beneficial bacteria in the intestine (Xiang et al., 2020). FMT has also been linked to an increase in beneficial bacterial species and a decrease in pro-inflammatory cytokines and markers of inflammation such as CRP and fecal calreticulin (Konturek et al., 2017).

#### Traditional Chinese medicine

Recently, traditional Chinese medicine (TCM) has shown promising results in treating diarrhea and is widely utilized in clinical practice. The ancient theoretical framework of Chinese medicine, which dates back over 2,000 years, has demonstrated both clinical and scientific efficacy in treating various diseases. Through extensive clinical practice, TCM has developed numerous effective and safe formulas, grounded in the principles of “Jun”-“Chen”-“Zuo”-“Envoy” (Yang et al., 2023). Due to the advantages of low side effects, long-lasting efficacy, and wide range of targets of action, the composition and mechanism of action of TCM have been extensively studied in modern times (Zhang et al., 2023b; Zhu et al., 2025). Herbal formulations and monomers in TCM can alleviate diarrhea symptoms through their anti-inflammatory, antioxidant, antimicrobial, and immune-boosting properties. The inflammatory signaling pathways associated with intestinal inflammation, along with the mechanisms of action of TCM formulas and monomers in addressing this inflammation, underscore the potential application of TCM in treating diarrheal diseases in animals (Table 5).

**TABLE 5:** The role of Chinese traditional medicine in relieving diarrhea.

Name	Mechanism	References
QJC (Chinese herbal formula composed of astragalus, astragalus and plantain herb)	Increasing the expression levels of PI3K and Akt, inhibiting the expression of phosphatase and tensin homolog (PTEN), and activating the PI3K-Akt signaling pathway alleviated the pathological changes in small intestinal tissues and improved the integrity of the intestinal barrier.	(Zhang et al., 2021)
HLJDD (Composed of 4 herbs: Cabis chinensis Franch, Phellodendron amurense Rupr, Gardenia jasminoides J. Ellis, and Scutellaria baicalensis Georgi).	HLJDD pretreatment maintains the level of CD44 in the renewal crypt cells, and the restoration of CD44 raises a certain level of Wnt signaling pathway activity to maintain rapid cell renewal during chemotherapy to repair the intestinal wall.	(Chan et al., 2020)
Improved Ren Shen Wu Mei Soup (MRWD)	Improves intestinal barrier function by promoting electrolyte transport, maintaining intestinal integrity, and inhibiting the TLR4/NF-κB signaling pathway.	(Guan et al., 2021)
SLBZP (poria mushroom and white artichoke powder)	The action of SLBZP is primarily linked to its anti-inflammatory, antioxidant, and immunomodulatory properties. Additionally, it contributes to the repair of damage to the intestinal mucosa, helps protect the intestinal mucosal barrier, encourages the migration of bone marrow-derived mesenchymal stem cells (BMSCs) to the colonic mucosa, influences specific signaling pathways, and supports the balance of gut microbiota.	(Chen et al., 2022)
JPY (Jianpiyintang)	JPY reduces GLP-1 expression, decreases ubiquitination and phosphorylation of NHE3, modulates NHE3 expression, improves ion transport in the intestinal epithelium, and ameliorates imbalances in electrolyte absorption.	(Ma et al., 2023)
LC, the main active compound of RD	Inhibition of intestinal caccs-mediated fluid secretion and TMEM16A-mediated intestinal motility	(Sun et al., 2019)
Ginger and heart laxative soup (SXD)	Significantly improved the diversity of gut microbiota and accelerated the recovery of gut microbiota. At the genus level, SXD significantly increased the relative abundance of *Anabaena* (*p* < 0.01) and decreased the relative abundance of *E. Coli* (*p* < 0.001). SXD significantly improved bile acid metabolism and amino acid metabolism.	(Zhang et al., 2023a)
Bupihewei soup (BPHW)	Significantly inhibited the expression of TLR-4, NF-κB, and inflammatory factors (including TNFα, IL-1β, and IL-6)	(Sun et al., 2019)
Pueraria lobata polysaccharide	Altered the gut microbiota, significantly increasing the relative abundance of beneficial bacteria and decreasing the relative abundance of pathogenic or diarrhea-associated bacteria. Increased fecal concentrations of isobutyric, propionic, and pantothenic acids; decreased levels of PC, arachidonic and docosahexaenoic acids.	(Shen et al., 2023)

## Conclusion

Diarrhea is closely associated with alterations in gut microbiota. Therefore, it is crucial to investigate how microbial communities detect pathogen infections to enhance our understanding of the pathogenesis of diarrhea. Furthermore, given that various probiotics possess distinct properties, future research should focus on further elucidating the specific mechanisms by which probiotics contribute to the rehabilitation of beneficial microbial colonies, thereby facilitating the clearance of diarrhea-causing pathogens.
